# Editorial: Gut microbiota: allied with livestock nutrition, health, and welfare

**DOI:** 10.3389/fvets.2024.1413671

**Published:** 2024-05-14

**Authors:** Balamuralikrishnan Balasubramanian, Wen-Chao Liu

**Affiliations:** ^1^Department of Food Science and Biotechnology, College of Life Science, Sejong University, Seoul, Republic of Korea; ^2^Department of Animal Science, College of Coastal Agricultural Sciences, Guangdong Ocean University, Zhanjiang, China

**Keywords:** gut health, natural resources, microbiota, nutrients, feed additives, livestock

## Abstract

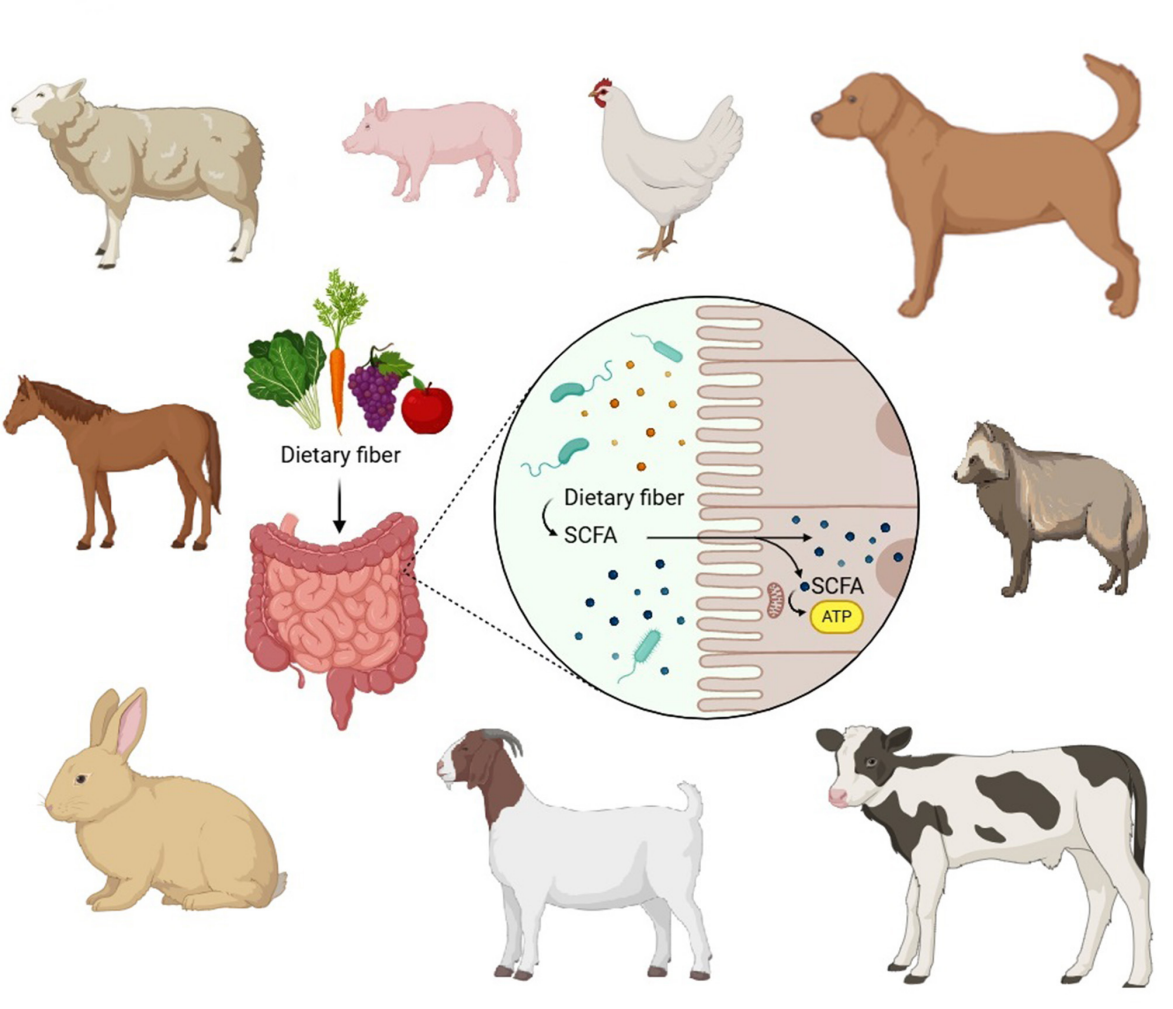

## Introduction

The majority of digestion and assimilation occur in the livestock's gastrointestinal tract. The availability of essential nutrients in a balanced diet is the key factor for successful animal production. The gut is associated with many microbiota that act as an extensive barrier, play an active role in immune development, and accelerate dietary challenges. Furthermore, the gut microbiome aids in communication at the cellular/tissue level and mediates the animal's overall metabolism. In short, the proper functioning of the gut is necessary to perform multiple functions to enhance the health, productivity, and sustainability of livestock farming. Thus, intestinal disorders can be minimized by understanding the role of the gut in animals.

From another perspective, several antibiotics are used to counteract the diseases and infections associated with intestinal disorders. However, a comprehensive approach to the use of probiotics, involving nutritional genetics and animal behavior, increases the possibility of resilience and robustness in animals. This lowers the rate of gut-related diseases and reduces the consumption of commercial drugs. However, clinical drugs will be used to treat other infections and diseases. In other words, the intestinal microbiome performs an important barrier and digestive role in the gut, which has been well evidenced in *in vivo* livestock models. Gut functionality and microbiome colonization are the triggers and supports for the immune system. Moreover, early interventions to strengthen gut health provide cues for overall livestock development. A wealth of research has demonstrated the complementary association between the gut microbiome, the immune system, and the brain. The gut microbiome also influences behavioral traits, for stress and anxiety. Overall, gut health is influenced by the diet, composition, and function of the GIT barrier with efficient digestion and assimilation factors, which in turn modulate the overall immune state of the animal. The microbiota aids in fermentation absorption, enhances immunity and growth, and improves host development. In addition, it regulates the stabilization of the gut environment and maintains the ruminal pH. Thus, the gut microbiota accelerates feed efficacy, and high-performance animals are vital objectives in livestock agriculture to meet the growing demand for animal products. In total, we received 16 articles that emphasize the critical role and significance of gut microbiota in growth, development, immune modulation, gut-brain barrier, digestion, feed utilization, and disease resistance (Graphical Abstract). This editorial aims to provide the essence of the research articles published in this domain to facilitate community stakeholders.

## Characterization and role of fecal microbiota in livestock farming

The microbiota is a specific microbial community that inhabits a particular setting within the animal's gut. Therefore, they serve as essential identification tools for understanding the insights of genetic traits across the specific breed or lineage characterization of the microbial community and aid in understanding the co-evolutionary relationships within the specific breed characters. The study by Carrillo Heredero et al. characterized the fecal microbiota composition of the *Bardigiano* horse and compared the data with the athlete breeds. Samples were identified using 16S rRNA sequencing. The results indicated that the *Bardigiano* breed showed prominent differences at the lower taxonomic levels. In addition, factors such as weight, origin, and breeding sites accelerated the microbiota composition. Moreover, a comparison with the athlete breed confirmed that environmental factors contribute to the microbial population, which is critical for safeguarding animal diversity, preserving animal health, and developing livestock farming.

Interestingly, differences in the gut microbiome and metabolism have been noted as the physical and pathological conditions of the host change The mechanism of association between the microbiome likely aids in the development of dairy cow health, which provides cues for the advancement of probiotics in cattle production. A recent study by Hsieh et al. investigated the gut microbiota and metabolome biomarkers in healthy and mastitis-lactating dairy cows. *In vitro* fermentation and cow-to-mouse fecal microbiota transplantation (FMT) were investigated. The results showed that *Ruminococcus flavefaciens* and *Bifidobacterium longum* subsp. *longum* rumen metabolites correlated with the healthy cow group. Inter-relationship similarity was verified by the upregulation of putrescine, xanthurenic acid, and pyridoxal in the mastitis ruminal fluid. These results evidenced the close alliance between the microbiome of dairy cows and their respective metabolites. The FMT confirmed the role of *R. flavefaciens* and *B. longum* subsp. *longum* in reducing mortality and improving the recovery of body weight loss. Adding organic and inorganic components to animal feeds has been shown to have significant growth-promoting properties. A recent study (Du et al.) reported that Copper (Cu) is an essential trace element for the growth of rabbits and has an influence on the intestinal microbiota and short-chain fatty acids (SCFAs) in growing rabbits. The cecal contents of the experimental rabbits (basal, inorganic Cu, and organic Cu fed) were investigated using 16S rDNA gene amplification sequencing and chromatography analysis. The relative abundance of *Rikenella Tissierella, Lachnospiraceae* NK3A20 group, *Enterococcus*, and *Paeniclostridium* was highest in the cupric citrate-fed group of rabbits, which was a factor in the lower incidence of diarrhea in these animals. The results revealed that the organic Cu-fed experimental groups had an abundance of *Rikenella* and *Enterococcus*.

## The potential of dietary supplementation to enhance the gut microbiota

The inappropriate usage of antibiotics in livestock and poultry has become an alarming and severe health problem. The impact of antibiotics has been observed as residues present in the egg; however, this can be overcome by adopting probiotics as feed additives to improve the health of breeders and their egg quality. In this context, a recent study reported the effect of β-mannanase and probiotics on the growth performance, serum levels, gut morphology, and egg quality traits of hens (Carvalho et al.). The β-mannanase and probiotic-supplemented feed improved egg-laying capacity in the hens by 11% and 7%, respectively. The combination effect of the (β-mannanase + probiotics) improved several yolk traits and serum biochemical parameters such as total cholesterol, uric acid, and triglycerides. Notable morphological variations in villus height and crypts were also reported, improving nutrient absorption levels (Carvalho et al.).

*Salmonella* is one of the most common infectious bacteria, causing severe infections in humans and livestock animals. However, *Salmonella enterica serovar Typhimurium* (ST) causes non-typhoidal infections and inflammation in the gut, disrupting the microbiome and leading to enterocolitis and dehydration in pigs (Yi et al.). Strengthening gut health through feed additives such as resistant starch (RS) increases the production of SCFAs in the intestinal tract and reduces gut inflammation. Weaned experimental pigs were treated with feed supplemented with raw potato starch (RPS), which resulted in better gut health and increased production of SCFAs. Histopathological lesions were reduced in the treated groups compared to the control group animals. The expression of IL-18 was low in the RPS-treated groups. However, Reg 3γ expression varied significantly across the different gut sites of the cecum and colon in both groups. Thus, the study concluded that RPS augmentation reduced inflammation and infection severity, preventing fecal shedding in ST-infected pigs (Yi et al.). Similarly, another study investigated the differences in the growth, biochemical, antioxidant, and gut microbiome of *Cyberlindnera jadinii*-supplemented feeds in raccoon dogs (Zhao et al.). The results reported improved immunity, gut health, growth development, and behavioral performance at the supplement concentration of 1 × 10^9^ CFU/g. Furthermore, the *C. jadinii*-treated groups exhibited improved average daily gain and a decreased feed-to-weight ratio (Zhao et al.).

Dang et al. investigated the dietary effect of tributyrin and anise mixture (TA) in weaned pigs. The experimental results showed that the TA mixture improved body weight, weight gain, average daily feed intake, and efficiency in pigs. Furthermore, noticeable improvements were observed in dry matter, crude protein, and energy digestibility, and jejunal villus height accelerated fecal microbial diversity. Populations of LAB strains such as *Lactobacillus reuteri, Lactobacillus amylovorus*, and *Clostridium butyricum* increased, improving digestibility and reducing ammonia emissions (Dang et al.). Feed augmentation of lambs with *Lemus chinensis* hay and *Alfalfa* hay was investigated, and observations on rumen microbiota populations and their metabolism were studied (Wang H. et al.). Increased carcass weight, loin-eye area, kidney weight, and abundance of the species *Fibrobacteres, Bacteroidetes*, and *Spirochaetes* were noted. Metabolomic approaches to several pathways were studied, while BF31, *Alistipe*s, *Faecalibacterium, Eggerthella*, and *Anaeroplasm*a were associated with growth and metabolic indices. The effects of growth and weight gain indices were evaluated in the Boer crossbred goats fed with selected commercial diets supplemented with *Hemarthria altissima, Pennisetum sinese*, and forage maize. Differences in the carcass traits and semi-eviscerated and eviscerated slaughter percentages were reported (Wu et al.). However, there was no significant difference in growth performance. The maize-fed animals exhibited abundant amino-acid content in the semimembranosus muscles. Microbiome analysis using 16S rRNA gene sequencing revealed the abundance of *Firmicutes, Bacteroidetes*, and *Proteobacteria* phyla. In particular, the *Rikenellaceae*_RC9 and *Oscillospiraceae*_UCG-005 correlated with the lipid metabolism and fatty acid composition, respectively. Therefore, the relative abundance of these rumen microbiota impacted the fattening, meat quality, and nutritional indices of goats (Wu et al.).

## The role of probiotics in accelerating the richness of the gut microbiome

The change in habitat from the inside of the uterus to the outside world requires additional nutrition and sustenance for calves. Developing immunity and digestive capacity plays a critical role in growth, survival, and gut health. In the study by Wang J. et al., it was demonstrated that the mode and composition of feed accelerated the impact on the development of the gut microbiome in newborn calves. According to the study, the Holstein bull calves were fed three types of diets for an experimental period of 80 days. The results showed that the SH group showed marked differences in the development of intestinal epithelium with goblet cells, and higher daily gain was observed with no pathogenicity or inflammation. Furthermore, improved digestibility and absorption impacted the immune status of the calves (10). Thus, strengthening the feed during pre-weaning is considered for the overall growth and development of calves. The gut microbiome of young animals includes a variety of bacteria colonized in the gut by maternal exposure, habitat, and feeding patterns.

To explore the entity of the microbiome, full-length sequencing in the rumen samples of Pastured yaks (in different age) was investigated by using 16S and 18S rRNA sequencing platforms to study bacterial and fungal communities (Yang et al.). The results showed that the bacterial diversity increased exponentially from the first to the eighth week of birth. However, the *Prevotella* species was the most abundant among all the other groups. Interestingly, the development of the fungal communities was observed at 90 days, which facilitated their growth and reproduction. Therefore, the study reported the occurrence of the rumen bacterial and fungal microbiota of Zhongdian yaks at different ages, which illuminated the potential changes of the main microflora aligned with the age and growth of yaks (Yang et al.).

In a similar work (Song et al.), the efficacy of the dietary stimbiotic (STB) supplement on growth performance, intestinal morphology, microbiota, and immune response in piglets challenged with Shiga toxigenic *E. coli* (STEC) was studied. The STB-supplemented diets decreased WBC, neutrophil, lymphocyte, and tumor necrosis factor-alpha expression levels and interleukin-6 levels. However, iliac villus height and claudin1 expression levels were found to be increased. STB supplementation increased the abundance of *Desulfobacterota* and *Fibrobacterota* to counteract the inflammatory response induced by *E. coli* in the challenged piglets (Song et al.). The feeds of pigs contain non-digestible carbohydrates such as cellulose. A recent study revealed that the microbiome is involved in the fermentation of non-digestible carbohydrates (NDC) such as resistant starch, xylo-oligosaccharide, and fructo-oligosaccharide in the large intestine of swine (Pandey et al.). However, inadequate enzymes make it difficult for swine to digest this component. Therefore, they depend on the gut microbiota for their degradation and energy utilization. Thus, a study reported identifying such microbiome involved in the fermentation of the NDC in the intestine of swine through next-generation high-throughput sequencing to provide insights into their metabolism-related and nutritional roles (Pandey et al.).

Similarly, weaned pigs were treated with a combination of *Lacticaseibacillus casei* and *Saccharomyces cerevisiae*, and growth, hematology, and immunological parameters were evaluated (Kim et al.). Adequate weight gain and feed utilization efficiency were increased in the treated group, while there were no marked differences in hematological and immune response parameters. Microbiota, such as the *Treponema* genus, were comparatively higher than *Lactobacillus* and *Roseburia* genera in the treated animals than in the control. Thus, the study provided cues to understand the association between gut microbiota and growth performance (Kim et al.). The effects of ammonia toxicity on the on-rumen microbiota and fermentation were investigated (Shen et al.) using the *in vitro* rumen fermentation technique. Correlation analysis revealed a negative association between free ammonia nitrogen (FAN) and the microbiome, *in vitro* rumen fermentation profile, total volatile fatty acid, and total ammonia nitrogen (TAN). Fluctuations in microbiome structure were observed as a function of TAN levels. Elevated TAN levels induced an abundance of *Firmicutes* and *Actinobacteria* but reduced the populations of *Fibrobacteres* and *Spirochaetes*. Thus, the study established that *in vitro* rumen fermentation by high ammonia is dependent on the rumen microbial communities, accelerating the pH dependence *in vitro* (Shen et al.).

A similar study demonstrated the effects of *Pediococcus acidilactici*, prebiotic lactulose, and their symbiotic combination on gut microbiota using 16S rRNA gene sequencing in weaned piglets challenged with STEC. *Prevotella, Roseburia*, and *Succinivibrio* were increased in the probiotic-fed groups, while the abundance of *Phascolarcto bacteria* was reduced in the challenged group. Thus, the study demonstrated that the administration of symbiotics improved intestinal health by modulating the gut microbiota in piglets (Guevarra et al.). Therefore, the overall research findings show that improving dietary practices with probiotic-rich sources provides effective cues for healthy livestock production. It may also serve as a reinforcing objective to enhance antibiotic-free animal products and augment gut microbiota modulation.

## Author contributions

BB: Conceptualization, Investigation, Validation, Visualization, Writing – original draft, Writing – review & editing. W-CL: Conceptualization, Supervision, Validation, Visualization, Writing – original draft, Writing – review & editing.

